# Numerical simulation of the flow and erosion behavior of exhaust gas and particles in polysilicon reduction furnace

**DOI:** 10.1038/s41598-020-58529-y

**Published:** 2020-02-05

**Authors:** Wang Qili, Jia Binbin, Yu Mingquan, He Min, Li Xiaochuan, Sridhar Komarneni

**Affiliations:** 1 Key Laboratory of Coal Processing and Efficient Utilization, Ministry of Education, China University of Mining and Technology, Xuzhou, 221116 P.R. China; 2 Hua Wei Chemical& Biological Engineering Co.,Lcd, Xi’an, Shanxi 710110 P.R. China; 3 School of Mechanical & Electrical Engineering, Xuzhou Institute of Technology, Xuzhou, Jiangsu 221008 P.R. China; 4 Department of Ecosystem Science and Management and Materials Research Institute, 204 Energy and the Environment Laboratory, The Pennsylvania State University, University Park, PA 16802 USA

**Keywords:** Chemical engineering, Computational science

## Abstract

In order to study the flow and erosion behavior of gas-solid exhaust in the polysilicon reduction furnace, the flow characteristics of exhaust gas and silicon particles were analyzed. The flow model and erosion model of exhaust gas and silicon particles were established based on the gas-solid flow theory and the erosion theory. The erosion and wear behavior of the gas-solid mixture in the flow passage pipeline were studied by numerical simulation. The results show that the wear and erosion from Nos. 1 to 8 regions at the bottom of the ring were caused by silicon particles colliding with high angle. The wear and erosion of 2 regions from Nos. 9 to 10 at the outside of the up azimuth on both sides of loop pipe outlets, 4 regions from Nos. 11 to 14 on the upper and lower wall of single furnace main channel were severely affected wear regions, which is caused by silicon particles with low angle and high velocity. Through comparative analysis, the erosion of upper wall of single furnace main channel is most serious. Increased gas velocity, particle concentration and particle size will exacerbate the erosion and wear rate of the pipeline in polysilicon reduction furnace, but the distribution and development of severe wear zone would not be affected significantly.

## Introduction

In the production of polysilicon, the improved Siemens process using SiHCl_3_ as the raw material and preparing high-purity silicon via H_2_ reduction, has been widely applied due to its high reliability and strong applicability for large-scale production. Polysilicon produced via this process accounts for more than 75% of the total production capacity of polysilicon around the world, and 95% of that in China^1,2^. The hydrogen reduction process of SiHCl_3_ is the key step of high-purity silicon preparation and a complex reaction process with both reduction and decomposition. There are some unreacted ingredients and products in the exhaust of this step. Since silicon particles are relatively hard, when they flow through the pipes of the exhaust system together with reducing exhaust, these particles will continuously scratch and erode the flow passage components. Thus, the safety of the exhaust system of polysilicon reduction furnace is seriously threatened, which becomes a potential safety hazard during polysilicon production.

The erosion caused by particles is one of the hot focuses in pipeline process industries. Many scholars such as Brown, Lee, Habit, Grant and Yao^3–7^ have conducted massive theoretical investigations and practical explorations on the erosion phenomenon and obtained favorable results. Finite element simulations are often used in conjunction with the Lagrangian and Eulerian method to study gas-solid two-phase flow and friction phenomena in pipes^8–10^. Gabriel^11^ utilized the Computational Fluid Dynamics(CFD) method and studied the erosion behavior of 90°elbow pipes in the petroleum industry. Deng^12^ studied the influence of solid particle concentration on the elbow erosion during pneumatic transmission, and the results showed that due to the shielding effect of particles, the erosion rate was reduced when the solid-phase concentration increased. Based on Euler continuity equation and erosion model, Joseph^13^ studied the erosion behavior of transport pipeline with 0.5 mm in diameter via numerical simulation. The results showed that hard particles and pipe status were the major causes of local erosion. In Lin’s research, the influence of erosion angle and elbow geometrical dimension on erosion was analyzed, and the gas-solid dual-phase flow simulation model was developed^14^. The thermo-fluid-solid coupling equations were developed by Du *et al*., and the flow filed, stress, and strain of multi-phase medium which flowed through pipe elbow were also studied with the help of FLUENT and ANSYS software^15^.

Based on the gas-solid two-phase flow theory and erosion theory, the flow phenomenon of gas-solid fluid in the exhaust outlet pipe of the reduction furnace in the polysilicon production was studied. By studying the collision and erosion process of silicon particles on the wall surface of the pipe, the local erosion of the exhaust pipe is predicted, so as to provide some support for the safe and reliable operation of the exhaust system in the reduction furnace.

## Experimental

The chemical composition and flow characteristics of the exhaust system are both very complicated. The gas-solid physical properties including composition, density, viscosity, particle dimension, morphology, and distribution in the exhaust system were determined. Sample analyses were carried out on deposited particles, which were collected from the outlet of the exhaust pipe of a running reduction furnace through a manual collection method using a cloth bag with many extremely tiny pores. X-ray diffraction (XRD) analysis of particles was conducted by using a D8 X-ray diffractor (Brooke). The results indicated that the deposited particles were almost all amorphous silicon, with a trace amount of crystalline silicon. According to the online composition analysis of on-site reduction furnace exhaust, the compositions and related proportions were SiHCl_3_ (11%), SiCl_4_ (7.5%), SiH_2_Cl_2_ (2%), H_2_ (78%) and HCl (1.5%), respectively. The density of silicon particles was measured to be 2330 kg/m^3^, based on the liquid phase displacement method. The exhaust density was calculated to be 2.668 kg/m^3^, via weighting the density of each component inside the pipe according to its molar ratio. The calculation method of natural gas mixture dynamic viscosity was utilized to calculate the pipe exhaust, and the exhaust viscosity was determined as 1.18 × 10^−5^ Pa·s. A BT-9300Z laser particle size analyzer was used to conduct grading analysis of silicon particles. The results implied that the minimum, maximum and average particle sizes of silicon particles were 2.87, 348.55 and 114.19 μm, respectively. All the parameters were shown in Table 1. Especially, the microscopic morphology of silicon particles has affection on the local erosion of the wall surface. The microscopic morphology was usually characterized by the sphericity degree defined by Wadell^16,17^.

1 $$\varphi =\frac{{S}_{V}}{S}$$

Where $\varphi$ is the coefficient of sphericity degree, ${S}_{v}$ is the surface area of equal volume sphere and $S$ is the surface area of the particle. Both ${S}_{v}$ and $S$ are obtained experimentally.

**Table 1:** Results of physical properties of gas and silicon particles in exhaust system^18^.

Parameters	Results
Molar percent of exhaust components	SiHCl_3_ (11%); SiCl_4_ (7.5%); H_2_ (78%); SiH_2_Cl_2_ (2%); HCl(1.5%)
Density of particles(kg/m^3^)	2325
Density of exhaust(kg/m^3^)	3.668
Viscosity of exhaust(Pa.s)	1.18 × 10^−5^
Average sizes of particles(μm)	*d*_min_ = 2.87; *d*_avg_ = 114.19; *d*_max_ = 348.55
Specific surface area of particles (m^2^/kg)	118.56
Distribution of particles	0–5 μm (10%); 5–200 μm (65%); >200 μm (25%)
Shape factor of particles	0.7–0.9

**Figure 1:**
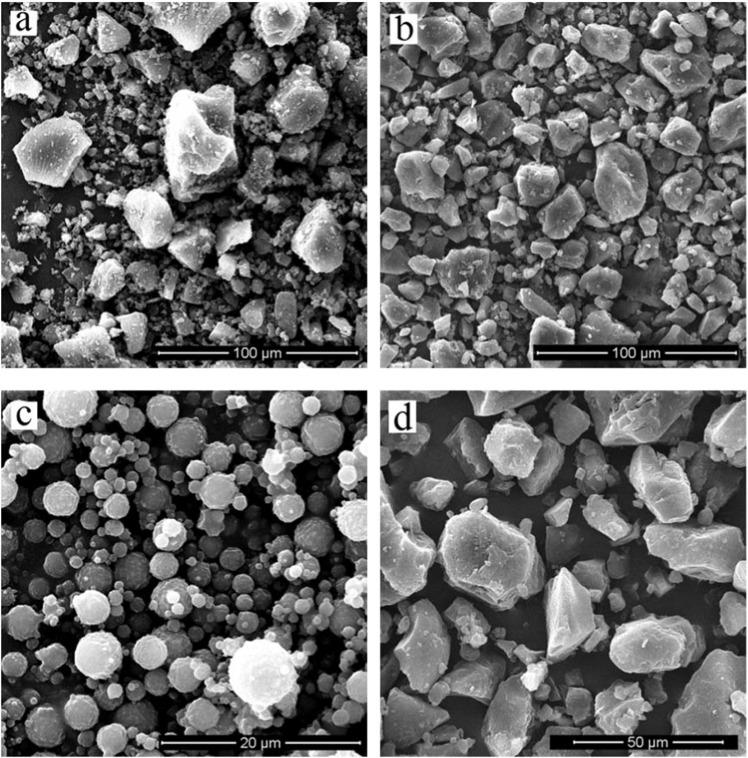
SEM morphologies for the silicon particles.

The scanning electron microscopy (SEM) images of the silicon particles of the exhaust system are clearly shown in Fig. 1. The overall appearances of the collected particles are clearly showed in Fig. 1(a,b). Figure 1(c) is the appearance of the particles after sieving according to a spherical shape, and the appearance of the other particles is shown in Fig. 1(d). It was found that the small and midium-sized particles were mostly spherical (Fig. 1(c)), while large-size particles presented features of strip and polygon (Fig. 1(d)). The shape coefficient of silicon particles was estimated to be 0.7–0.9.

## Gas-solid Flow And Erosion Behavior Of Exhaust System

### Flow analysis of reducing exhaust and silicon particles

Eulerian-Eulerian method and Eulerian-Lagrangian method are usually used for the simulation of gas-solid flow fields. The characteristic of the former is the two-fluid model, in which particle phases are also treated as continuous fluids. The control equations for continuous phases are used here for calculations. This method is suitable for two-phase flow problems with similar particle sizes and relatively large particle-phase volume fractions. It obtains continuous phase flow field features under the Euler coordinate system, while solving the particle phase motion under the Lagrange coordinate system. The two-way coupling effect between two phases is also considered^19,20^. Thus, the statistical description of macroscopic motion of particles in the flow field is achieved. This method is often used in the treatment of dilute phase motion. Hence, it is necessary to determine the flow state of silicon particles in reducing exhaust and select a suitable method to describe the mixed flow of exhaust and silicon particles.

According to the value of particle-phase volume fraction $(\phi )$, the gas-solid two-phase flow can be divided into the dense flow and dilute flow. Usually, the gas-solid flow under the condition of $\phi  < 10 \%$ is treated as a dilute flow. According to the on-site statistics of deposited silicon particles in the exhaust system and the load of the exhaust flow, the percentage of solid silicon particles in the exhaust was estimated to be lower than 1%. Hence, the flow of reducing exhaust carrying silicon particles was dilute phase motion, and it was reasonable to use the Eulerian-Lagrangian method to describe this two-phase flow phenomena. Also, the two-way coupling calculation mode was selected, considering the constant exchange of momentum between silicon particles and exhaust during flow. The flow of continuous phase (exhaust) was described using the transport model under the Euler framework. Based on the Discrete Phase Model (DPM) model under the Lagrangian coordinate, the motion trail of discrete phase (silicon particles) was obtained. Moreover, the coupling solution of exhaust flow and silicon particle motion was realized by considering the two-phase momentum exchange.

### Flow model of reducing exhaust

During gas-solid two-phase flow in a pipe, the reducing exhaust is a continuous phase, and its Reynolds number is calculated to be greater than 4000, indicating that it is a normal turbulent flow. Due to the influence of exhaust viscosity and pipe wall non-slip condition, the turbulent exhaust flow inside pipe is divided into a turbulent core region and a near-wall flow region. A significant difference exists between the flow features of them. Hence, it is necessary to conduct analysis on the turbulent core region, near-wall flow region and silicon particle motion.

The exhaust turbulent flow core refers to the region which is far away from pipe wall, and its flow is fully developed. The Reynolds stress in the flow field is greater than the viscous stress. The time-averaged speed distributes uniformly. This region is the dominant zone for silicon particles to acquire kinetic energy, which directly decides the motion track of silicon particles and affects the wall surface impact features. The flow analysis of the exhaust turbulent flow core region is of crucial importance to the erosion behavior of exhaust system. The mean motion of turbulence is a major concern in engineering, while the influence of random fluctuations is ignored. The averaging method is used to study its macro flow characteristics. The continuity equation, motion equation, and energy equation are constructed as follows^21,22^.

2 $$\left\{\begin{array}{rcl}\frac{\partial {\bar{u}}_{i}}{\partial {X}_{i}} & = & 0\\ \frac{\partial {\bar{u}}_{i}}{\partial t}+{\bar{u}}_{j}\frac{\partial {\bar{u}}_{i}}{\partial {X}_{j}} & = & -\frac{1}{{\rho }_{g}}\frac{\partial \bar{p}}{\partial {X}_{j}}+\frac{1}{{\rho }_{g}}\frac{\partial }{\partial {X}_{j}}(\mu \frac{\partial {\bar{u}}_{i}}{\partial {X}_{j}}-{\rho }_{g}\overline{u{\text{'}}_{i}u{\text{'}}_{j}})\\ \frac{\partial }{\partial t}(\frac{{\bar{u}}_{i}{\bar{u}}_{i}}{2})+\frac{\partial }{\partial {X}_{j}}[{u}_{j}(\frac{\bar{p}}{{\rho }_{g}}+\frac{{\bar{u}}_{i}{\bar{u}}_{i}}{2})] & = & \begin{array}{c}-(-\overline{{u}_{i}^{\text{'}}{u}_{j}^{\text{'}}})\frac{\partial {\bar{u}}_{i}}{\partial {X}_{j}}+\frac{\partial }{\partial {X}_{j}}(-\overline{u{\text{'}}_{i}u{\text{'}}_{j}}.{\bar{u}}_{i})+\\ V\frac{\partial }{\partial {X}_{j}}[{\bar{u}}_{i}(\frac{\partial {\bar{u}}_{i}}{\partial {X}_{j}}+\frac{\partial {\bar{u}}_{j}}{\partial {X}_{i}})]-V(\frac{\partial {\bar{u}}_{i}}{\partial {X}_{j}}+\frac{\partial {\bar{u}}_{j}}{\partial {X}_{i}})\frac{\partial {\bar{u}}_{i}}{\partial {X}_{j}}\end{array}\end{array}\right.$$

A turbulence model should be introduced to solve the closed problem of the basic equation group. The standard ${\rm{\kappa }}-{\rm{\varepsilon }}$ model is suitable to be constructed based on the vortex viscosity hypothesis which reflects the relationship between Reynold stress and flow field^23^. The turbulence energy ${\rm{\kappa }}$ and turbulence kinetic energy dissipation rate ${\rm{\varepsilon }}$ are introduced to describe the spatial scale and time scale of turbulence. The turbulence viscosity is determined to be $\,{\mu }_{i}$. Furthermore, the Reynold stress is calculated to make the flow basic equation group closed.

For the gas-solid flow in pipe, silicon particles need to pass through the near-wall exhaust flow barrier, before they impact the wall surface. Due to viscosity and vortex, it is unavoidable for silicon particles to exchange energy with the near-wall flow, resulting in the change of motion state. The wall function method is usually used in the analysis of near-wall flow. The flow condition is simplified and a near-wall flow field fitting the turbulence center flow based on the semi-empirical formula is established. There is no special requirement for the discretization of the near-wall space. Instead, the nodes in the initial end into the logarithm layer are needed to be set, and the conditions corresponding to the flow in the turbulence core region is needed to be established. This method is suitable for describing the near-wall viscous bottom layer flow of reducing furnace exhaust. Its speed distribution function, turbulence energy, and turbulence dissipation rate are described as follows.

3 $$\{\begin{array}{rcl}{u}^{+} & = & \{\begin{array}{ll}{y}^{+} & ({y}^{+}\le 11.63)\\ \frac{1}{\kappa }\,\mathrm{ln}\,(E{y}^{+}) & ({y}^{+} > 11.63)\end{array}\\ {G}_{k} & \approx  & {\tau }_{w}\frac{{\tau }_{w}}{\kappa {\rho }_{g}{{C}_{\mu }}^{1/4}{k}^{1/2}\Delta y}\,\\ \varepsilon  & = & \frac{{{C}_{\mu }}^{3/4}{k}^{3/2}}{\kappa \Delta y}\,\\ {y}^{+} & = & \frac{\Delta y}{V}\sqrt{\frac{{\tau }_{w}}{{\rho }_{g}}}\end{array}$$

Where u^+^ is the velocity of the near wall flow region, y^+^ is the distance between the near wall flow region and the boundary, both u^+^ and y^+^ are dimensionless parameters, $\,{\rm{\kappa }}$ is Von Carmen constant, $\,{u}_{\tau }$ is the friction speed of the wall, $\bigtriangleup {\rm{y}}$ is the distance from the fluid to wall, ${\tau }_{\omega }$ is the shear stress of the wall and $E$ is the empirical constant, equal to 9.8.

### Erosion model of silicon particle impacting wall surface

The material erosion failure is mainly controlled by environmental factors, abrasive performances, and target material properties. The variability and coupling effect of these factors result in different erosion mechanisms. The construction of erosion mathematical model contains three aspects: (1) constructing the control equation for energy exchange relationship caused by the collision between particles and material on the macro level; (2) constructing the failure model reflecting the internal microstructure damage on the micro level; (3) constructing the interaction between external macro collision and internal microstructure damage. Tabakoff, Fu and Zhang^24–26^ established the physical equation of erosion failure, by combining the macro cause of erosion with a micro-mechanism based on energy conservation and mechanical actions.

During the recycling of reducing exhaust, the pipe is made of stainless steel. Silicon particles have hardness like that of SiO_2_ particles. The erosion model proposed by Oka and Yoshida is more suitable for the erosion of flow passage components in the exhaust system^27–29^. This model focuses on the material failure behavior under particles’ perpendicular impact and constructs a mathematical model containing influencing factors including impact speed, the diameter and shape of the particle, and target material hardness, as shown in Eq. 4. This model also considers the erosion test results of materials including aluminum, copper, carbon steel, and stainless steel. Based on these tests, related parameters are acquired, making this model relatively consummated.

4 $$\left\{\begin{array}{ccc}\Delta E & = & g(\alpha ){E}_{90}\\ g(\alpha ) & = & {(\sin \alpha )}^{{n}_{1}}{(1+(1-(\sin \alpha ))}^{{n}_{2}}\\ {E}_{90} & = & K{({H}_{V})}^{{k}_{1}}{[\frac{{u}_{p}}{{u}_{{p}^{\text{'}}}}]}^{{k}_{2}}\,{[\frac{{d}_{p}}{{d}_{{p}^{\text{'}}}}]}^{{k}_{3}}\\ {n}_{1} & = & {S}_{1}{({H}_{V})}^{{q}_{1}}\,\\ {n}_{2} & = & {S}_{2}{({H}_{V})}^{{q}_{2}}\end{array}\right.$$

Where *E*_90_ is the target erosion rate at 90° collision angle(mm^3^ • kg^−1^), $\alpha$ is the collision angle, $g(\alpha )$ is the ratio of the amount of wear caused by the particles to the amount of vertical impact wear at any collision angle, which reflects the mechanism of repeated plastic deformation and shear failure caused by particle collision. $H{\rm{\nu }}$ is the initial Vickers hardness of the target, $K$, ${k}_{1}$, ${k}_{2}$ and ${k}_{3}$ are empirical constants, determined by particle properties and material hardness, ${u}_{p^{\prime} }$ and ${d}_{p^{\prime} }$ are the impact velocity and particle diameter in the erosion experiment. ${s}_{1}$, ${s}_{2}$, ${q}_{1}$ and ${q}_{2}$ are empirical constants that reflect the particle properties, with the values being 0.71, 2.4, 0.14, 0.94 and 4, respectively^27,28^. The flow characteristics and particles properties of exhaust system in the reduction furnace are very similar to the erosion models of Oka and Yoshida. Therefore, the erosion model can be used to simulate the erosion behavior in the exhaust gas system of the reduction furnace and some of the main parameters have been given by Oka and Yoshida^27–29^.

### Numerical simulation results and discussion

**Figure 2:**
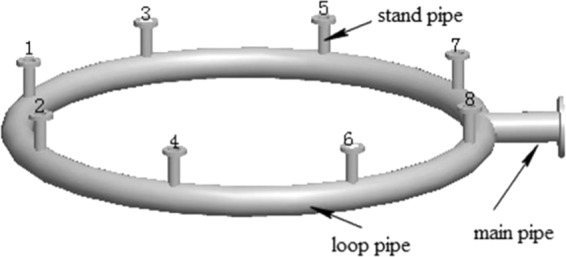
Structure of exhaust outlet pipe.

As shown in Fig. 2, the exhaust outlet pipe of the reducing furnace consists of eight standpipes with a diameter of 80 mm and a height of 200 mm, abase-plate loop pipe with a pipe diameter of 200 mm and a ring diameter of 3000 mm, and a main pipe with a diameter of 200 mm and a length of 400 mm. The gas and particles discharged from the furnace are collected to the exhaust loop pipe of the reducing furnace and finally flow into the main pipe. This section of pipe plays both the roles of exhaust collection and transport. Thus, it is the first to be affected by the continuous impact of silicon particles and becomes the weak point of safety in the exhaust system. Hence, the study of the erosion behavior of exhaust system in polysilicon reducing furnace focuses on this structure. According to the data from industrial application, the simulation conditions are determined as follows: the exhaust velocity of 5, 7, 9, 11 and 13 m/s, the silicon particle diameter of 50, 80, 115, 150, and 200 μm, and the silicon particle concentration in exhaust of 12, 17, 22, 27, and 32 g/Nm^3^. The system has an average working condition of the exhaust velocity of 9 m/s, the particle diameter of 115 μm, and the silicon particle concentration of 22 g/Nm^3^, which is the real data from the current working condition of industrial field. Especially, the gas and silicon particles in the exhaust system have the same flow velocity, regardless extremely small speed differences. The ANSYS and FLUENT programs are selected for simulation and the SIMPLE algorithm is used. In order to improve the discretization efficiency, the nonstructural mesh algorithm with high adaptability is used to divide the mesh by “Tet/Hybrid” technology. The fluid flow regions are mainly composed of tetrahedral elements, and some of the combined regions are hexahedral and cone elements. The grid skew rate was controlled within 0.8, and the grids with skew rates lower than 0.4 accounted for 91% of the total number of grids. Finally, 1.13 × 10^6^ discrete elements were obtained.

According to Oka and Yoshida’s theory and model, “mm^3^ • kg^−1^” is used as the unit of volume erosion rate of per unit mass caused by particle collision, and “kg • s^−1^” is used as the unit of mass flow of particles^27–29^. However, because of a more direct understanding, “mm • s^−1^” is commonly used as the velocity erosion rate in engineering applications and finite element simulation projects, which is the ratio of the mass erosion rate generated by the particles of per mass flow to the area of the erosion region. “mm • s^−1^” describes the erosion rate in the normal direction of the contact surface when particles collide with the wall surface, which is used as the unit of erosion rate in this simulation research.

### Flow and erosion features of exhaust outlet pipe under the average working condition

**Figure 3:**
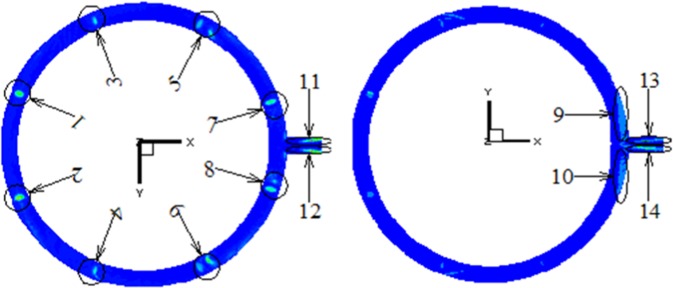
Distribution map of local erosion of exhaust outlet pipe.

Under the average working condition of *μ*_0_ = 9 m/s, *d*_*p*_ = 115 μm and *C*_*p*_ = 22 g/Nm^3^, the local erosion distribution of the exhaust outlet pipe is shown in Fig. 3. Each erosion region is labeled for further discussion. 14 key erosion regions are labeled in the whole exhaust outlet pipe, with Nos. 1–10 regions for the base-plate loop pipe and 11–14 regions for the main pipe. Since the flow region presents complete spatial symmetry, the Nos. 1, 3, 5, 7, and 9 regions of the base-plate loop pipe, are corresponding regions of Nos. 2, 4, 6, 8, and 10 regions. Similarly, Nos. 11 and 13 regions of the main pipe are also corresponding regions of the Nos. 12 and 14.

**Figure 4:**
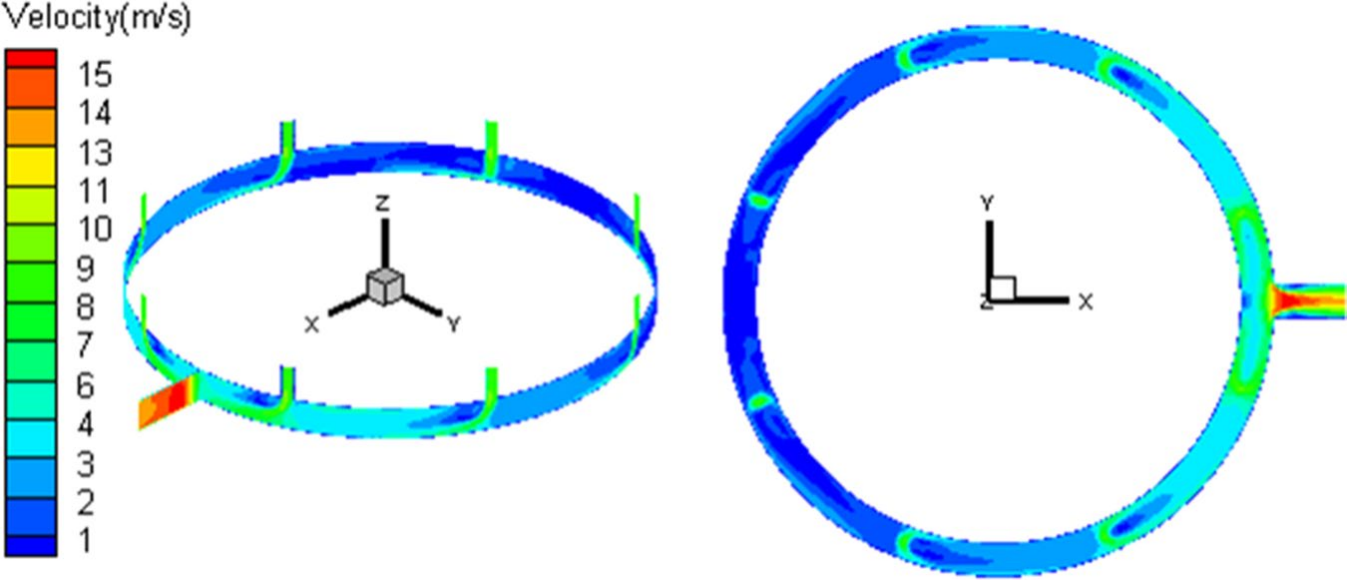
Distribution of velocity of exhaust outlet pipe.

A Z-direction center section is constructed in the flow region. The speed distribution nephogram under the average working condition is shown in Fig. 4. According to the figure, the loop pipe flow presents following features: Firstly, the closer to the flow outlet, the greater the flow speed is. Since the gas flow increases with the collecting flow of stand pipes, while the flow region is unchanged, the flow speed increases obviously. Second, the stand pipe flow shifts more seriously in regions closer to the outlet. Since the upstream flow speed in the loop pipe is small, its influence on the downstream shift of stand pipe flow is limited. Meanwhile, the flow speed of loop pipe downstream increases, the shift degree of afflux gas enhances. Since the gas flow in loop pipe is constrained by the flow passage, under the influence of centrifugal effect, the gas flow speed on the outside is greater than that inside. Due to the combined influence of loop flow and collecting flow initial motion, vortex flow locally occurs in the inlet downstream. With the increase of loop flow, the influence of vortex flow becomes stronger. Thus, the closer to the outlet, the more disorder the flow field is. In the main pipe section, the exhaust transits from circular constraint flow to flatflow space and the flow state changes sharply, leading to vortex flow in local regions of the inlet end. Afterward, the flow field expands along the outlet end of the main pipe. Under the influence of no-slip wall, the speed in the center of the flow field is high and decreases to two sides.

**Figure 5:**
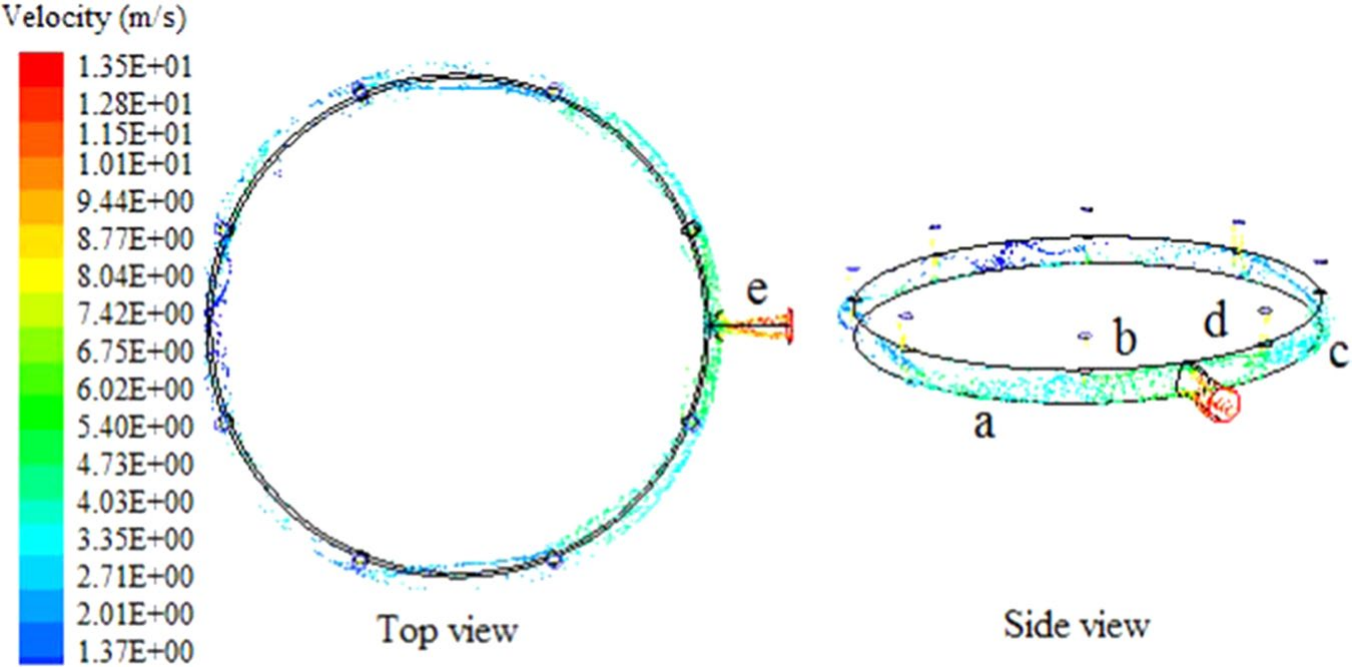
Trajectory of silicon particles in exhaust outlet pipe.

By tracking silicon particles in the outlet pipe, the particle kinematic trajectory can be obtained. As shown in Fig. 5, the branch collecting flow particles directly impact the inlet wall. Due to the influence of circulation flow, the motion of collecting flow particles shifts. The closer to the outlet of loop pipe, the stronger the circulation flow, and the more seriously the particle motion trajectory shifts. After collision with the wall, collecting flow particles bounce and show upward helical motion under the influence of circulation flow, which is shown from region *a* to region *b* and from region *c* to region *d* in Fig. 5. At the outlet of loop pipe, the helical motion develops towards the outer surface and upward, due to the influence of outlet gas entrainment. The particle concentration in the outer region of the upside increases. During the loop pipe flow pass developing into the main pipe space, gas carrying particles enters the main pipe with a certain angle. As shown in the region *e* of Fig. 5, the particle motion presents an obvious development tendency of diffusion.

**Figure 6:**
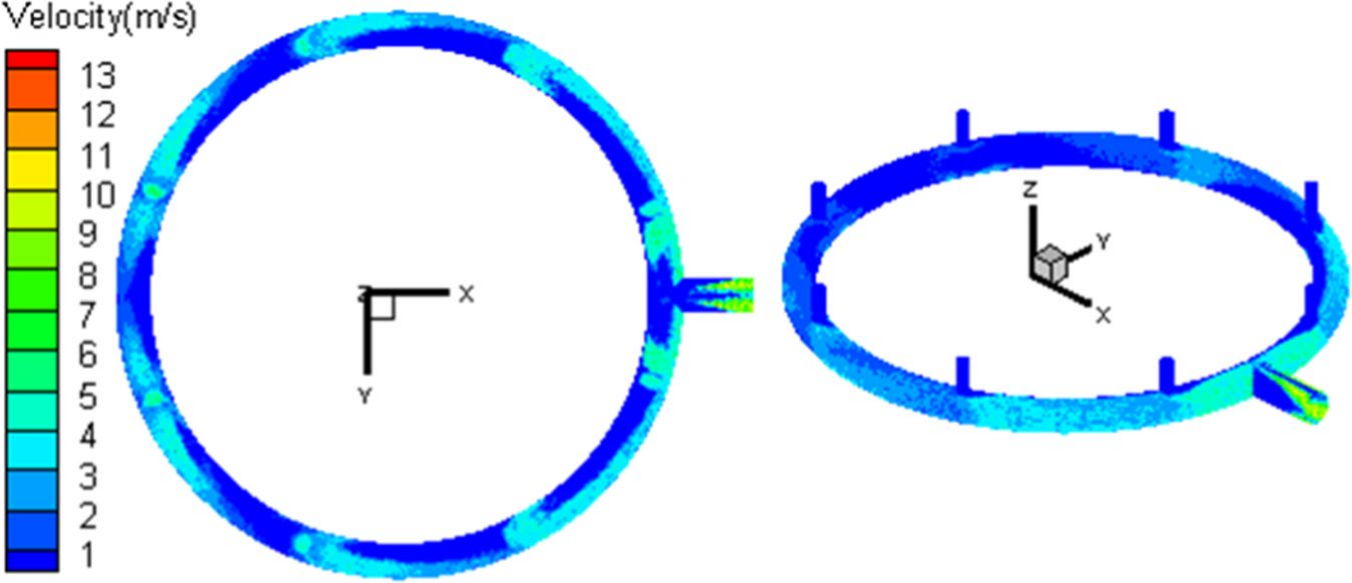
Velocity of the particles collide with outlet pipe.

**Figure 7:**
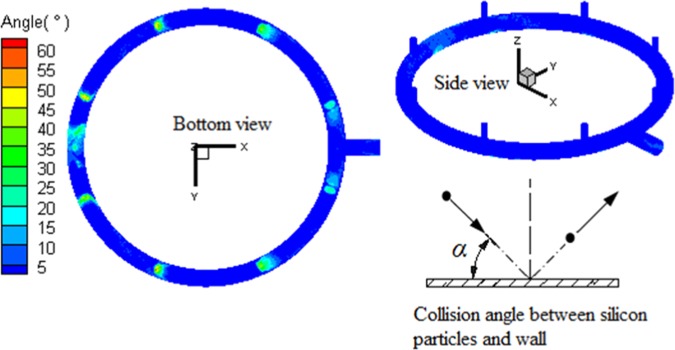
Angle of the particles collide with outlet pipe.

The statistics results of angle and velocity distribution during the collision between silicon particles and wall are demostrated in Figs. 6 and 7. The particle collision angles in Nos. 1–8 regions of loop pipe are greater than those in Nos. 9–10 regions of loop pipe and Nos. 11–14 regions of main pipe. Since the former locates in the front of the stand pipe, the collecting flow particles present a tendency of frontal collision to the local wall. The collision angle (α) is large, between 90° and 120°. Meanwhile, local collision in the latter is caused by particles moving with gas flow. It presents a near-wall slide motion, with low impact angles between 0 and 5°. Under the influence of circulation flow, collecting flow particles shift from the front of the inlet. With the circulation flow becomes stronger, the shift increases, and the frontal collision tendency decreases. Thus, the collision angles of particles in Nos. 1–8 regions decrease with the distance to the outlet end decreasing. Since the closer to the outlet, the more gas flows into the pipe. When the gas flow speed increases in fixed passageway, the particle velocity also increases. This leads to the collision velocity of Nos. 11–14 regions of the main pipe to be higher than that in Nos. 1–10 regions of the loop pipe. Hence, the erosion of Nos. 1–8 regions is caused by the high-angle direct collision, while the erosion in Nos. 9–14 regions is caused by the low-angle and high-speed erosion.

**Figure 8:**
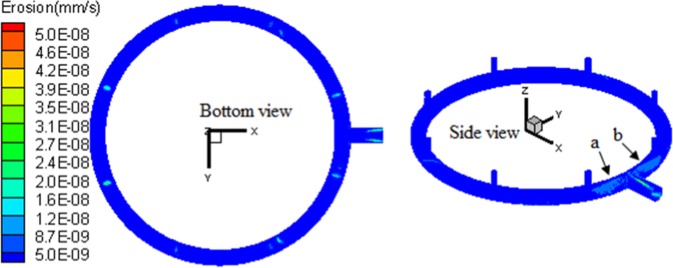
Nephogram of local erosion of exhaust outlet pipe.

The nephograms of particle collision angle, collision velocity, and erosion distribution in the local region of the outlet pipe are shown in Figs. 6–8, respectively. Serious erosion regions in the base-plate loop pipe contain two parts. The first is the region directly opposite the entrance of the Nos. 1–8 stand pipes. This region presents small-angle rotational shift along loop pipe center line and helical development along the outer edge of curved surface, when the distance between loop pipe and outlet decreases. The second is the upper outer edge regions (region *a* and region *b* in Fig. 8) around the loop pipe outlet. The serious erosion region of the main pipe section develops from the upper and lower surfaces to the outlet.

The causes of local erosion features in the outlet pipe are summarized as follows. (1) Nos. 1–8 regions are high-frequency collision zones, which are preferentially collided by silicon particles flowing into the loop pipe, resulting in serious erosion. (2) Under the influence of in-pipe gas flow, particles vertically flowing into the loop pipe shift with the flow. The closer to the outlet, the greater the gas flow speed, and the greater is the particle motion shift. Hence, with the decrease of distance to the outlet, the angles between Nos. 1–10 regions and the stand pipe front region increase. (3) After colliding in Nos. 1–8 regions, particles bounce back. Due to the influence of circulation flow, the particle trajectory after rebound presents a helical motion. The particles further collide the wall surface. Hence, the Nos. 1–8 erosion regions present a helical development tendency. (4) At the outlet, particles are entrained by outlet gas flow and the helical trajectory experiences a shift towards the upper outer side of the outlet. Thus, the Nos. 9 and 10 erosion regions around the center line are generated. (5) Due to the influence of circulation flow, particles enter the main pipe with certain tangential angles and move with the gas flow. The flow field features of particle response to main pipe widen towards the outlet. The impacting regions gradually experience related changes.

### Influences of working condition parameters on the outlet pipe erosion

**Figure 9:**
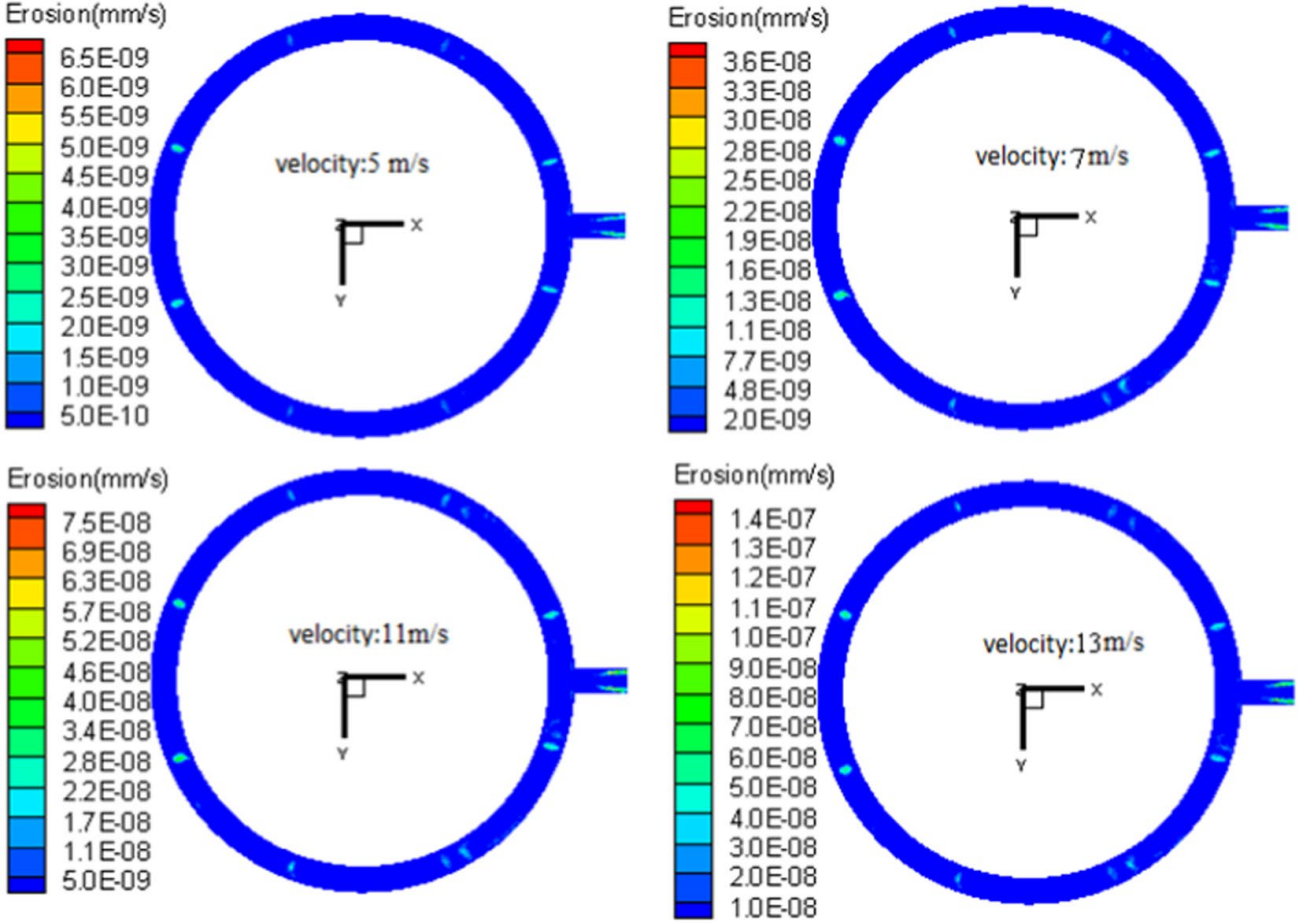
Nephogram of local erosion of outlet pipe with different exhaust velocity under the condition of *dp* = 115 μm, *C*_*p*_ = 22 g/Nm^3^.

Under the condition that the silicon particle size and inlet concentration are kept constant, by changing the exhaust flow velocity, the influence of exhaust velocity on the outlet pipe erosion was investigated and the results are shown in Fig. 9. With the increase of exhaust velocity, the erosion rate of each load region demonstrates an increase. However, under single-factor conditions, by increasing the exhaust velocity, the turbulent of flow field also increases. Silicon particles obtain more energy via interaction with the fluid. From a macroscopic view, the particle speed and kinetic energy increase and hence, the potential erosion capacity is increased. Therefore, more kinetic energy affects the collision region during collision, and aggravates the erosion degree of material. In addition, with increasing exhaust velocity, the quantity of silicon particles carried by exhaust is also increased, and more silicon particles take part in the local collision with flow passage pipe. Thus, the erosion condition is further worsened. However, during the increase of exhaust velocity, the distribution and development regularity of seriously eroded regions change little.

**Figure 10:**
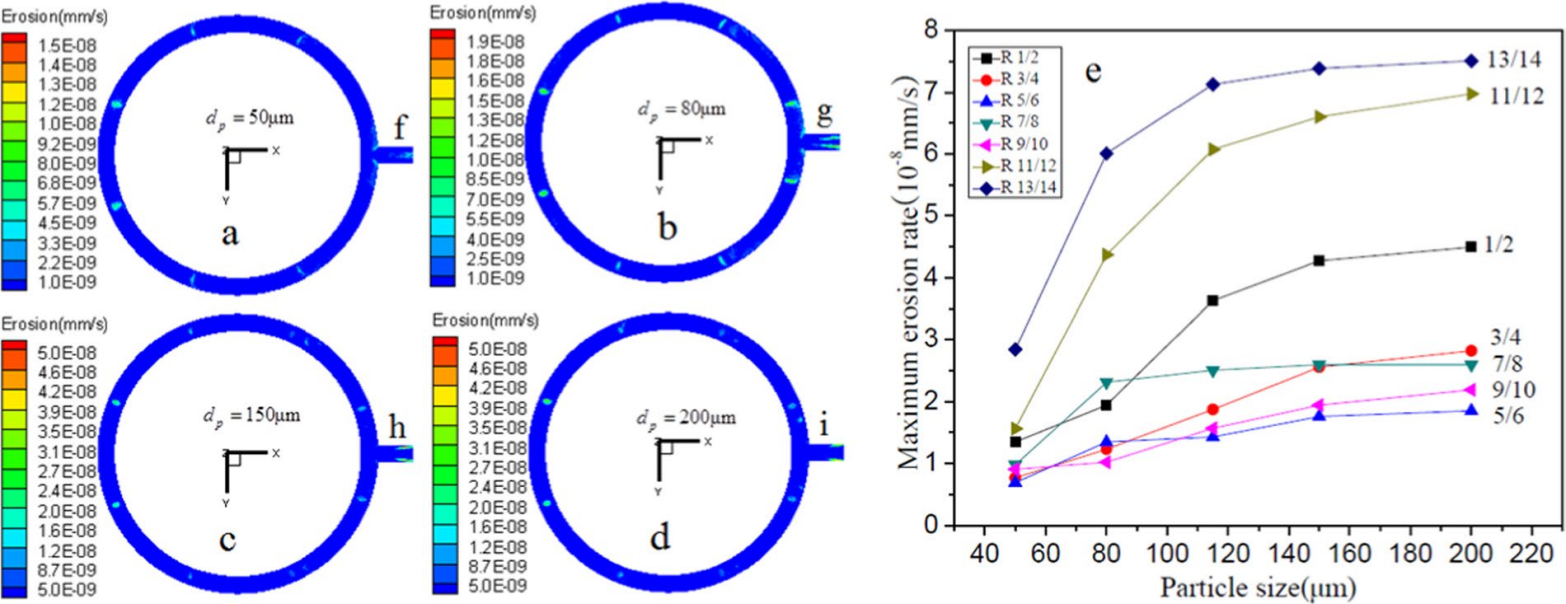
Nephogram of local erosion of exhaust outlet pipe with different silicon particles diameter under the condition of *μ*_0_ = 9 m/s, *C*_*p*_ = 22 g/Nm^3^. (**a**–**d**) Are the nephograms at *dp* = 50 μm, 80 μm, 150 μm, 200 μm, respectively. (**e**) Is the relationship between the maximum erosion rate and the particle size in different regions. (**f–i**) Are the regions in main pipe at different particle diameters.

Under the condition that the exhaust velocity and inlet concentration are kept constant, by changing the silicon particle diameter, the influence of particle size on the outlet pipe erosion is investigated and the results are shown in Fig. 10. It implies that particles with different diameter show different erosion characteristics in different regions. Firstly, the erosion rate in all regions shows different increasing trends with increasing particle size. The erosion rate increases with the increase of silicon particle diameter because larger diameter particles have higher mass and kinetic energy, which results in greater collision and erosion. The increase in Nos. 1–10 regions is slight, and the increase in the of Nos. 11–14 regions is significant. Secondly, under the condition of constant particle concentration, the smaller particle diameter means the larger number of particles, and an increase in particle diameter means a reduction in the number of particles in the airflow and a lower collision frequency. So the erosion area is reduced because of the increasing particle diameters, which is clearly shown in Nos. 1–2 regions with different particle diameters (Fig. 10). Consequently, duo to the combined effect of increasing particle diameter and decreasing particle number, the collision between the silicon particles and the tube results in an increase in erosion rate, but a decrease in erosion regions.

However, a special case is that the strongest erosion and the largest erosion area of the of Nos. 7–8 regions occurs at *dp* = 80 μm. According to the nephogram of local erosion in Fig. 10(a–d) and the maximum of erosion rate for Nos. 7–8 regions in Fig. 10(e), some difference from other regions is shown that when the particle diameter reaches 80 μm, even if the particle size continues to increase to 200 μm, the maximum erosion rate remains almost unchanged, but the number of particles is significantly reduced. The possible reason is that when *dp* = 80 μm, the airflow is the most turbulent, and the particles have the worst following with the airflow. So, the increased dispersion of particles increases the contact area with the wall in the pipe, resulting in the largest erosion area. On the other hand, with the increase of the particle diameter and mass, the chaos in airflow causes stronger internal particle collisions and more kinetic energy loss, most of the increased kinetic energy due to mass increase is consumed by internal collisions between particles, resulting in no significant increase in the erosion rate.

Besides, it is also found that with the increase of particle size, the inertia and gravitational force of particles are also increased. Due to the change of particle motion features, local collision regions shift, leading to the variation of serious erosion regions. Since small particles have small inertia and strong ability to migrate with flow, during the circular passageway develops into straight passageway, the particle motion cuts into the main pipe space with small angles approximately along the flow line. Thus, the collision regions are distributed with small angles from the center line, which is demonstrated in regions *f* and *h* in Fig. 10(a,b). In comparison, as shown in regions *g* and *i* in Fig. 10(c,d), the inertia of large-size silicon particles is great and has a strong ability to maintain its circumferential flow tendency during transition flow. In the end, large-size particles enter the main pipe with large angles along the tangential direction. Thus, the collision regions develop with large angles from the center line.

**Figure 11:**
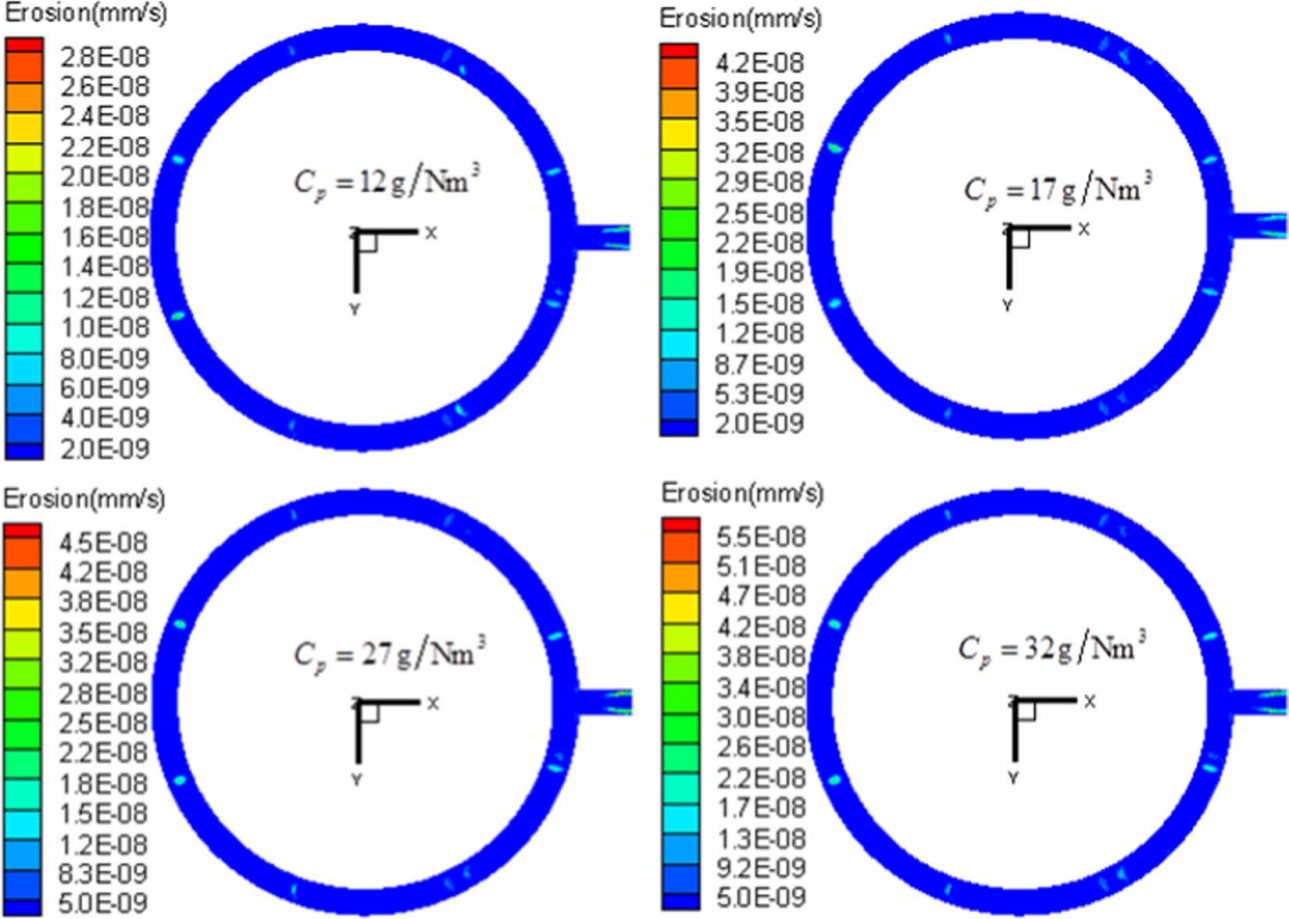
Nephogram of local erosion of exhaust outlet pipe under different silicon particles concentration under the condition of *μ*_*0*_ = 9 m/s, *d*_*p*_ = 115 μm.

Under the condition that the silicon particle diameter and exhaust velocity are kept constant, by changing the silicon particle concentration (*C*_*p*_ = 12, 17, 22, 27, 32 g/Nm^3^), the influence of particle concentration on the outlet pipe erosion is investigated. The results from Fig. 11 indicate that, with the increase of silicon particle concentration, the erosion rate of all local regions increases slightly. With the increase of silicon particle concentration, the number of local particles flowing through the pipe in unit time rises. Particles involving local collision increase and the collision frequency also increase. Hence, the surface erosion rate also rises. However, when the silicon particle concentration increases, the serious erosion regions are almost unchanged, and the development regularity of each local erosion region keeps unchanged.

## Conclusions

1. The exhaust system of the polysilicon reduction furnace consists of many kinds of gases and solid-phase particles. The gases are mainly SiHCl_3_, H_2_, and SiCl_4_, with a density of 3.668 kg/m^3^ and a viscosity of 1.18 × 10^−5^ Pa·s. The solid-phase particles are almost all amorphous silicon, with a density of 2.330 × 10^3^ kg/m^3^. The particle size ranges from 2.87 μm to 348.55 μm, with an average particle size of 114.19 μm. The shape of particles is mainly spherical, while large-size silicon particles present shapes like strips and polygons. The shape coefficient of silicon particles is between 0.7 and 0.9.
2. The volume fraction of solid-phases in the reducing exhaust is lower than 1%, and its Reynolds number is greater than 4000. Hence, the reducing exhaust belongs to dilute and turbulent flow. Among the 14 regions, Nos. 1–8 regions are the high-angle direct collision regions, among which, regions in the front of stand pipe inlet experience serious erosion; the Nos. 9–14 regions are caused by low-angle and high-speed erosion, with high-frequency collision regions formed in the upside region of the outlet in loop pipe. Thus, serious erosion is induced. The erosion of the main pipe is more serious than that of the loop pipe. The ultimate value of wall surface erosion rate in the upside of the main pipe is the largest among all the regions studied.
3. Working condition parameters have significant influences on the erosion behavior. When the other parameters are unchanged, the increase of the single parameter will increase the local wear rate of the pipeline. With the increase of exhaust velocity, both the quality and speed of silicon particles flowing through the pipe increase. More silicon particles collide the wall surface of the pipe with higher velocity, and the erosion further worsens. The erosion to materials aggravates, but the distribution and development regularity of serious erosion regions change little. The increase of silicon particle size leads to the increase of kinetic energy. While colliding the wall surface, more energy conversion takes place, and the erosion to materials also increases. The increase of particle concentration in exhaust flow improves the number of particles flowing through the pipe. With the number of particles involved in erosion and increased collision, the collision frequency and erosion rate are also increased.

## References

[1] Tian L, Gang X, Yu XH (2011). Research Status and Development Trend of Poly Silicon Production. Yunnan Metall..

[2] Jiang X, Zhou WH, Cheng HM (2013). Analysis and Forecast of Polysilicon Industry in China. Adv. Mater. Ind..

[3] Brown GJ (2002). Erosion prediction in slurry pipeline tee-junctions. App. Math. Model..

[4] Lee BE, Tu JY, Fletcher CAJ (2002). On numerical modeling of particle-wall impaction in relation to erosion prediction: Eulerian versus lagrangian method. Wear..

[5] Bozzini B, Ricottim ME, Boniardim M, Mele C (2003). Evaluation of erosion corrosion in multiphase flow via CFD and experimental analysis. Wear..

[6] Luo K, Liu XY, Jin J, Cen KF (2004). Numerical study of the ribbed duct anti-erosion efficiency in gas solid flows. China Soc. Elec. Eng..

[7] Yao J, Chen LH, Fan JR, Cen KF (2002). Numerical simulation of a new method for protecting bends from erosion in gas-particle flow. China Soc. Elec. Eng..

[8] Habib MA, Mansour RB, Badr HM (2008). Erosion and penetration rates of a pipe protruded in a sudden contraction. Comput. Fluids..

[9] Liu CW, Mao JR, Yu MZ (1999). Analysis of Gas Solid Two Phase Flow and Erosion in a 90°Curved Duct. J. Xi’an Jiaotong Univ..

[10] Zhang Y, Zhou W, Sun ZQ (2011). Numerical Simulation of Scouring Erosion Characteristics for Gas-solid two phase Flow in Pipes. Met. Mate. Metall. Eng..

[11] Gabriel CP, Francisco JS, Diego AMM (2014). Numerical prediction of the erosion due to particles in elbows. Powder Technol..

[12] Deng T, Chaudhry AR, Patel M (2005). Effect of Particle concentration on erosion rate of mild steel bends in a pneumatic conveyor. Wear..

[13] Achebo, J. I. Computational analysis of erosion wear rate in a pipeline using the drift flux models based on eulerian continuum equations. Proceedings of the World Congress on Engineering, pp. 719–721, (2009).

[14] Lin N, Lan HQ, Cui Y (2013). Effects of Incidence Angle and Geometry of Elbows on the Erosion. Sci.Technol. Eng..

[15] Ren, X. Z. *Multiphase Pipeline 9*0 *Degrees Elbow Erosion Stress Analysis*. Northeast Petroleum Unive. Press, China, 25–50, (2015).

[16] Wadell H (1932). Volume, shape, and roundness of rock particles. J. Geol..

[17] Liu EJ, Cashman KV, Rust AC (2015). Optimising shape analysis to quantify volcanic ash morphology. GeoResJ..

[18] Jia, B. B. *Erosion Research of Exhaust System of Polysilicon Reduction Furnace*. Chinese Univ. of Mining and Techno. Press. China, 56–79, (2015).

[19] Cen, K. F. & Fan, J. R. *Theory and Calculation of Gas- solid Multiphase Flow in Engineering*. Zhejiang Univ. Press, China, 74–89 (1990).

[20] Shi, X. G., Xu, X. C. & Feng, J. K. The Analysis of forces on particles moving in turbulent flow. *J. Eng. Thermophys*. **10**, 320–325(1989).

[21] Wang, F. J. *Computational Fluid Dynamics Analysis-Software Principles and Applications*. Tsinghua Univ. Press, China, 126–146, (2004).

[22] Grant G, Tabakoff W (1975). Erosion prediction in turbo machinery resulting from environmental solid particles. J. Aircraft..

[23] Wang XZ, Jiang ZA, Wang SW (2007). Numerical simulation of distribution regularities of dust concentration during the ventilation process of coal roadway driving. J. China Coal. Soc..

[24] Zhang J (1994). Simulation of the strongly swirling gas-particle turbulent flow by stochastic trajectory model. Acta. Mech. Sinica..

[25] Tabakoff W, Kotwal R, Hamed A (1979). Erosion study of different materials affected by coal ash particles. Wear..

[26] Lin, F. Y. *Wear principle and anti-wear technology*. Science Press, China, 136–201, (1993).

[27] Oka YI, Yoshida T (2005). Practical estimation of erosion damage caused by solid particle impact. Part1: effects of impact parameters on a predictive equation. Wear..

[28] Oka YI, Yoshida T (2005). Practical estimation of erosion damage caused by solid particle impact. Part2: Mechanical properties of materials directly associated with erosion damage. Wear..

[29] Oka YI, Mihara S, Yoshida T (2009). Impact-angle dependence and estimation of erosion damage to ceramic materials caused by solid particle impact. Wear..

